# Novel Mutations in *pncA* Gene of Pyrazinamide Resistant Clinical Isolates of *Mycobacterium tuberculosis*

**DOI:** 10.3390/scipharm86020015

**Published:** 2018-04-16

**Authors:** Manijeh Kahbazi, Hossein Sarmadian, Azam Ahmadi, Farshideh Didgar, Maryam Sadrnia, Toktam Poolad, Mohammad Arjomandzadegan

**Affiliations:** 1 Infectious Diseases Research Center (IDRC), Arak University of Medical Sciences, Arak, 3819693345, I.R. of Iran; Dr.kahbazi@arakmu.ac.ir (M.K.); dr.sarmadian@arakmu.ac.ir (H.S.); ahmadia22@yahoo.com (A.A.); dr.didgar@arakmu.ac.ir (F.D.); t.poolad@yahoo.com (T.P.); 2 Department of Pediatrics, School of Medicine, Arak University of Medical Sciences, Arak, Iran; 3 Department of Infectious Diseases, Arak University of Medical Sciences, Arak, Iran; 4 Department of Biology, Payame Noor University, 19395-4697, Tehran, I.R. of Iran; msadrnia@yahoo.com; 5 Department of Microbiology, School of Medicine, Arak University of Medical Sciences, Arak, Iran

**Keywords:** *Mycobacterium tuberculosis*, pyrazinamide, mutation, polymerase chain reaction, sequencing

## Abstract

In clinical isolates of *Mycobacterium tuberculosis* (MTB), resistance to pyrazinamide occurs by mutations in any positions of the *pncA* gene (NC_000962.3) especially in nucleotides 359 and 374. In this study we examined the *pncA* gene sequence in clinical isolates of MTB. Genomic DNA of 33 clinical isolates of MTB was extracted by the Chelex100 method. The polymerase chain reactions (PCR) were performed using specific primers for amplification of 744 bp amplicon comprising the coding sequences (CDS) of the *pncA* gene. PCR products were sequenced by an automated sequencing Bioscience system. Additionally, semi Nested-allele specific (sNASP) and polymerase chain reaction-restriction fragment length polymorphism (PCR-RFLP) methods were carried out for verification of probable mutations in nucleotides 359 and 374. Sequencing results showed that from 33 MTB clinical isolates, nine pyrazinamide-resistant isolates have mutations. Furthermore, no mutation was detected in 24 susceptible strains in the entire 561 bp of the *pncA* gene. Moreover, new mutations of G→A at position 3 of the *pncA* gene were identified in some of the resistant isolates. Results showed that the sNASP method could detect mutations in nucleotide 359 and 374 of the *pncA* gene, but the PCR-RFLP method by the SacII enzyme could not detect these mutations. In conclusion, the identification of new mutations in the *pncA* gene confirmed the probable occurrence of mutations in any nucleotides of the *pncA* gene sequence in resistant isolates of MTB.

## 1. Introduction

It was estimated that in 2012, 8.6 million people developed tuberculosis (TB) and 1.3 million died from the disease [1]. This annual fatality has turned it into a major health problem in most countries of the world. The significance of this issue has been more obvious in the case of occurrence of the higher resistance of tuberculoses to a number of drugs, multidrug-resistance (MDR) and extensively drug-resistance (XDR) that have been reported in countries around the world [2].

MDR or resistant to several drugs is the type of bacillus that is resistant to at least two drugs of Isoniazid and Rifampin [2]. Due to the incidence of XDR tuberculosis disease, a rising number of fatalities and failures to cure tuberculosis have become more important in the fight against this disease [1,2]. Furthermore, the increasing time and expenses of treatments have converted the possibility of the prevalence of these resistant strains of tuberculosis to a serious threat of the world that should be controlled.

Contrary to the numerous studies in other countries, only a few have been conducted in Iran. Pyrazinamide is one of the first-line drugs used for treatment which is a bacteriostatic drug that together with isoniazid acts as a quick bactericidal agent for *Mycobacterium tuberculosis* (MTB).

Similar to isoniazid, pyrazinamide is a synthetic derivative of nicotinamide that has been used for the treatment of tuberculosis since 1952 [3]. This bactericide compound is used to exterminate the proliferating bacilli. Application of this drug would decrease the processing of treatment from nine to 12 months to less than six months. This conventional anti-tuberculosis drug exterminates a colony of tuberculosis bacilli in the acidic pH environment that are not abolished by other anti-tuberculosis drugs. This first-line drug for the treatment of tuberculosis only affects MTB during active inflammation in an acidic environment [4].

Mutation in the coding nucleotide sequence (CDS or opening reading frame, ORF) of the *pncA* gene is the cause of resistance to the pyrazinamide drug. This gene encodes a pyrazinamidase enzyme. The hot-spots of occurrences of mutations are at nucleotides 359 and 374 of *pncA* CDS.

In the present study, we evaluated the coding sequence of the *pncA* gene sequence in clinical resistant and sensitive isolates of MTB. 

## 2. Material and Methods 

In this study, 33 clinical isolates of MTB were provided from the Infectious Diseases Research Center (IDRC), Arak University of Medical Sciences, Iran, that had already been phenotypically determined to be resistant or susceptible to pyrazinamide. 

Grown MTB colonies in solid Lowenstein-Jensen culture medium were used for extraction through the Chelex100 kit (Sigma, St. Louis, MO, USA) [5]. 

**Figure 1:**
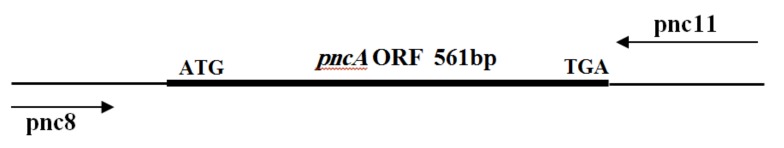
Schematic view of the position of the used primers (arrows). The size of the amplicon was 744 bp, 561 bp from ORF and 183 bp from downstream and upstream of this gene.

The primer sequences used were acquired from other studies [6]. The used primers bind upstream of the first nucleotide and downstream of the last nucleotide of the *pncA* gene [6]. These primers (Table 1) are used in the amplification reaction by a thermo-cycler machine (Eppendorf, Germany) to produce a 744 bp fragment (Figure 1). A polymerase chain reaction (PCR) is optimized according to temperature melting and the concentrations of primers.

PCR reactions using 40 ng of DNA, 2.5 microliters of 10× buffer with mg (Cinnagen, Tehran, Iran), 10 pmol of each primers, 10 mM deoxynucleoside triphosphate (dNTP) (Cinnagen) and 1 unit of Taq polymerase enzyme (Cinnagen) were performed in a total volume of 25 µL. The touchdown temperature protocol was used in PCR including these steps: (1) denaturing initial: 94 °C for 5 min. (2) 94 °C for 30 s, 68 °C for 30 s (decreasing 1 °C 10 cycles), 72 °C for 30 s and 20 cycles of 94 °C for 30 s, 58 °C for 30 s, 72 °C for 30 s, (3) final extension: 72 °C for 8 min.

**Figure 2:**
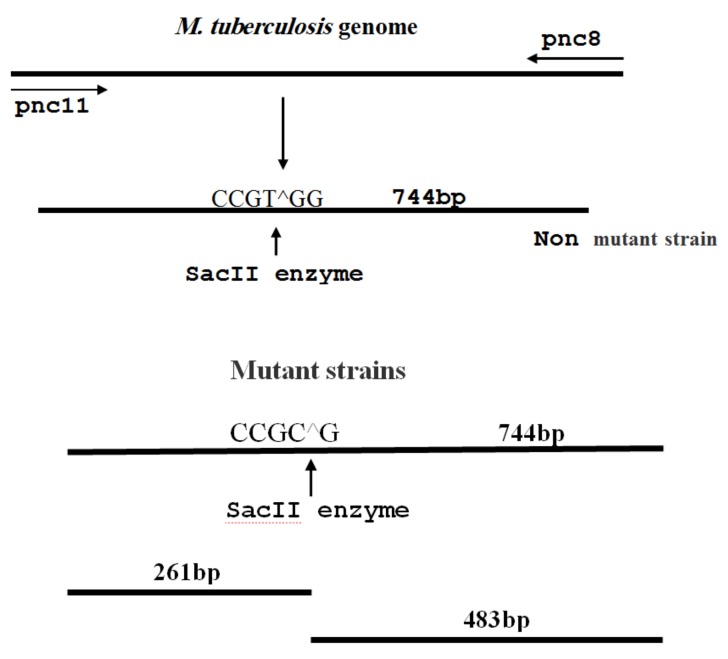
Schematic view of digestion of 744 bp fragment by the SacII enzyme. After digestion by enzyme, the 744 bp fragment produces 261 bp and 483 bp fragments in the mutant strains.

The restriction fragment length polymorphism (RFLP) method was used to identify mutations in the position of 359. Therefore, after amplification of the 744 bp fragment, based on Perdigao’s study in 2008 [7], the SacII enzyme was used to identify mutations in nucleotide 359. Restriction site of this enzyme is (5′-CCGC″GG-3′), which in the event of mutation at this point, a restriction place will be provided for this enzyme and it can practically identify the mutant form. The position of identifying this enzyme in the mutant form and expected bands were determined with Genetyx software (Genetyx, Win5.1 software, Japan). Production of 483 bp and 261 bp bands confirmed the occurrence of mutation (Figure 2).

In order to perform the digestion reaction, 10 µg of PCR produced from pnc-8 and pnc-11 primers, 2 µL10× buffer, 1 unit of enzyme and 18 µL of distilled water were mixed. Then, the resultant mixture was initially placed at 37 °C for 4 h and then at 65 °C for 20 min to inactivate the enzyme.

### 2.1. (S-NASP) Semi Nested-Allele Specific PCR Method 

**Figure 3:**
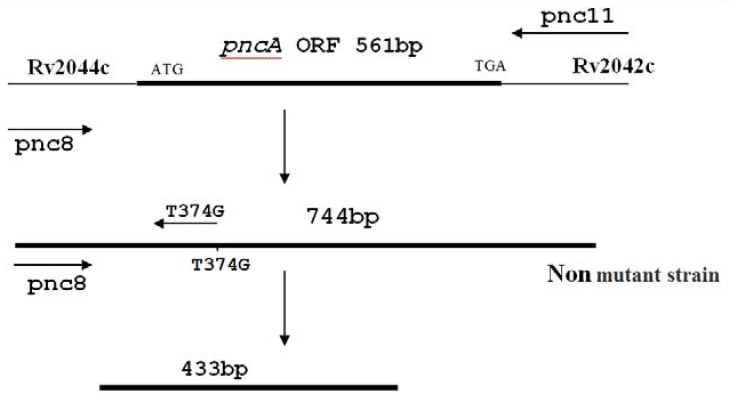
Detection of mutation using primer 374. When mutation occurs in nucleotide 374 (T→G), primer will not be able to detect mutations and amplification will not be realized. Creation of bands by primer 374 confirms the absence of mutation in this nucleotide.

**Figure 4:**
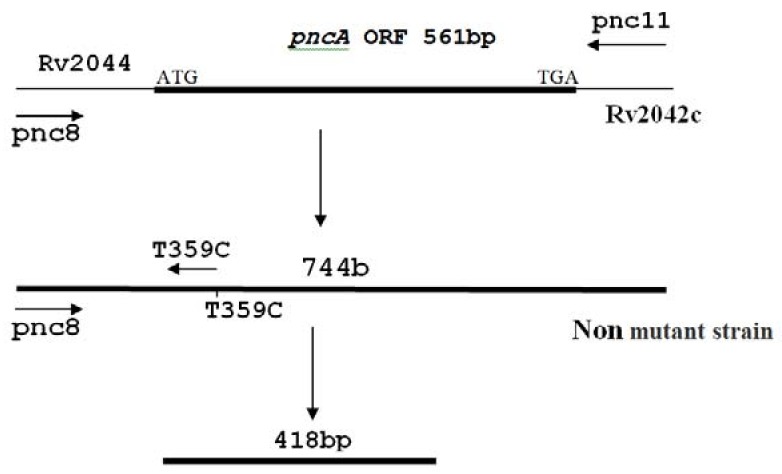
Detection of mutation using primer 359. When mutation occurs in nucleotide 359 (T→C), primer will not be able to detect mutations and duplication will not be realized. Creation of bands by primer 359 confirms the absence of mutation in this nucleotide.

In the semi nested-allele specific PCR (S-NASP) method, those 744 bp amplicons were used as a template. Then, in PCR, one of the main primers (pnc-8 or pnc-11) was applied with primers that have been designed for the accurate detection of the wild type status of the 359 or 374 nucleotide (allele-primers, T359C-pnc or T374G-pnc). To determine mutation using primer 374 (T374G-pnc): Creation of a band using primer 374 shows the absence of mutation at this nucleotide (Figure 3).

(B) determination of mutation using primer 359 (T359C-pnc): Creation of band using primer 359 showed the absence of mutation at this nucleotide (Figure 4).

Sequences of primers are listed in Table 1.

PCR Program was designed for sNASP as follows: (1) initial denaturing: 94 °C for 5 min (2) 35 cycles: Denaturation: 94 °C for 1 min, Annealing: 61 °C for 1 min, Extension: 72 °C for 40 s (3) final extension: 72 °C for 10 min. PCR conducted for a volume of 25 µL as follows: 1.5 µL of PCR product, 2.5 µL of 10× buffer containing mg, 2.5 µL of 10 pmol each primer pnc-8 primer and primer 347 (or pnc-8 and primer 359), 1 µLof 10 mM dNTP, 0.9 µL of Taq polymerase, and 14.1 µL of sterile distilled water.

### 2.2. Electrophoresis 

Amplified fragments loaded on 1% agarose gel (Cinnagen) and then stained with the safe-stain (Sinaclon- Tehran- Iran) were analyzed with UV light, using the transilluminator device (Quantum, San Jose, CA, USA).

### 2.3. Sequencing

Sequencing of produced amplicons was conducted for the determination of possible mutations. In this method, the 744 bp amplicons were sequenced with specific primers. This sequencing method is based on Sanger (Sanger Institute, Hinxton, Cambrige, UK) and uses the labeled nucleotide. Then, PCR products were purified from the gel, and sent by mediated Sinaclon Company for the accomplishment Source Bioscience Company of England.

## 3. Results

**Figure 5:**
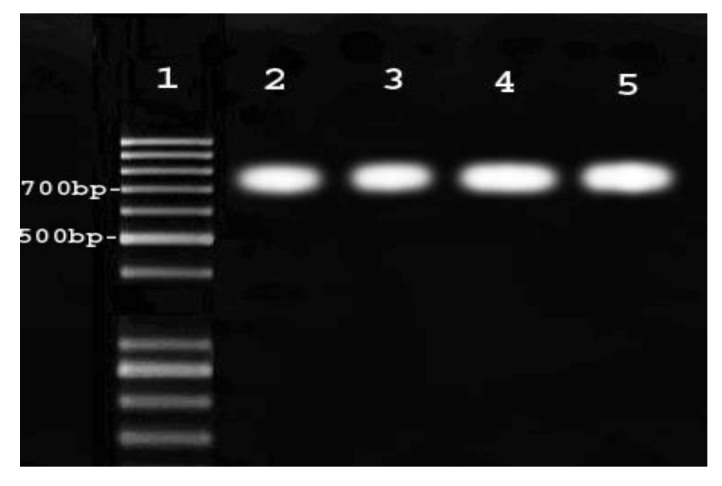
This figure showed results from the used primers pnc11 and pnc8. The resulting band shows that the function of primers is performed correctly.

**Figure 6:**
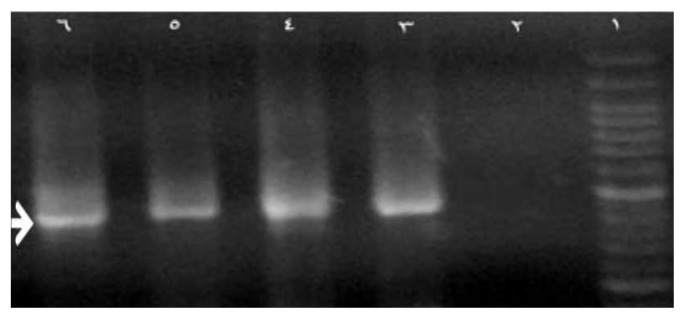
Semi nested-allele specific (sNASP) method: primers 374 on 744 bp PCR product in non-mutated strains.

**Figure 7:**
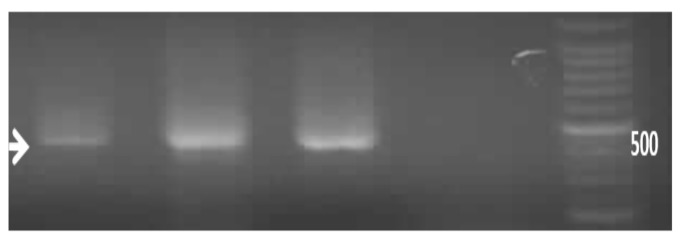
sNASP method: primers 359 on 744 bp PCR product in non-mutated strains.

Pnc11 and pnc8 primers were able to produce the 744 bp fragment. The resulting bands are shown in Figure 5, Figure 6 and Figure 7, indicating the proper function and determination of an appropriate amplification reaction.

In the RFLP method, given that this enzyme detects and digests the mutant sequence, the lack of digestion by the enzyme is thus due to the lack of a consistent sequence. Therefore, it was determined that the enzymes are not applicable for the identification of mutations. Probably, in the examined samples, no identification by the enzyme was observed due to the low frequency of mutations in nucleotide 359, or it can be concluded that the examined samples exhibited no mutation at nucleotide 359.

**Figure 8:**
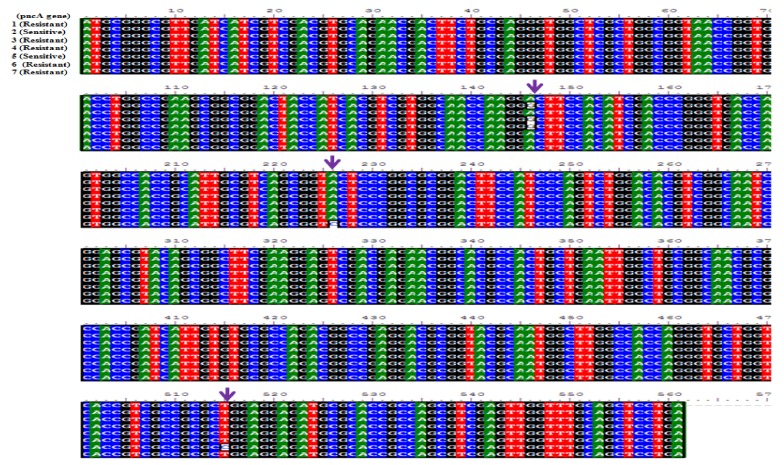
Results of sequencing of *pncA* sequence in some of the Sensitive (samples 2 and 6) and Resistant (samples of 2, 4, 5, 7, 8) isolates in the present study that were analyzed by —Bioedit software (BLOSUM62). The locations that are indicated by arrows show the mutation positions.

The results obtained from sequencing of the 744 bp fragment by Mega4, Chromas, and Bioedit software (Mega4 software version 4.0, Tempe, AZ, USA) were compared to sequences with no mutations in the gene bank. Some of these results are illustrated in Figure 8. 

Susceptible clinical isolates lacked mutation in nucleotides 359 and 374. In addition to the aforesaid mutations, we discovered new mutations not found in other literature by sequencing, such as G3A and T410G. Figure 8 shows that T would transform to C in the case of mutation in nucleotide 359, as specified by the sequencing of fragment 744 bp.

Results obtained from the sequencing of some sensitive strains are presented in Table 2 which depicts the phenotype-genotype adaptation of these strains.

## 4. Discussion

*Mycobacterium tuberculosis* (MTB) is the main cause of the infectious disease of tuberculosis. This disease may be cured by antibiotics in the case of timely diagnosis while the treatment procedure would be disrupted if a mutation exists in the genome of the bacteria. Drug resistance occurs due to genetic mutation at different genes in MTB [3,4]. Imperfect or wrong treatment would allow the drug-resistant bacilli to transform into the prevalent strain in the body of the infected person while the sensitive bacilli are exterminated due to the consumption of anti-tuberculosis drugs but resistant mutants would become proliferated as a result of defective treatments, turning into prevalent strains in the body of the patient. Unfortunately, this is the feature that not only causes the prescribed standard short-term treatment regime to not work for those infected with drug-resistant bacilli, but also develops resistance to a wider range of anti-tuberculosis drugs [2,6]. Pyrazinamide is a first-line drug for the treatment of tuberculosis and resistance to this drug is due to mutation in *pncA*. Quick diagnosis of mutations through sequencing contributes to the diagnosis of resistance to antibiotic bacteria [8,9]. 

The length of CDS of *pncA* is 561 bp. In order to examine the entire sequence coding, the primers were designed such that all the nucleotide sequences of this gene would be proliferated and result in 744 long amplicons (Figure 7). Sequencing of proliferated products has been used to accurately diagnose the drug-resistance in *M. tuberculosis*. This is used as a standard reference for the diagnosis of mutations and is nowadays performed using automatic sequence devices. Gold standard sequencing is a molecular method of DNA sequencing that may be used as an indicator. A total of 33 strains were sequenced in this research after PCR; all of the pyrazinamid resistant strains were XDR. Their resistance came from mutation in the coding sequence of the *pncA* gene. All nine resistant strains had mutation at *pncA* gene [8,9,10,11]. All nine strains showed various mutations at the *pncA*, which is considered as resistance to pyrazinamide. According to the results of other research and those of the current project, any variation in the *pncA* gene would lead to resistance against pyrazinamide in these bacteria. Somoskovi (2007) found one mutation in the nucleotide −11 *pncA* promoter as a result of a study on six resistant bacteria [12]. His results were verified by those of Lee (2001) [9] and Marttila (1999) [11]. Perdigao (2008) [7] proved mutation in nucleotide two of six resistant samples. He found 11 mutations in nucleotide 374. Sheen (2009) [13] discovered mutation in nucleotide 143 of *pncA* among three resistant samples and one sensitive sample. Bacro (2006) [14] stated that the results of sequencing support the previous findings indicating mutations in the *pncA* gene of many pyrazinamide-resistant mycobacterium strains. He attributes the main mechanism of pyrazinamide-resistance in isolated mycobacterium of Brazil to alteration in the gene and 22 different mutations in the *pncA* sequence. Bacro showed that 45.5% of mutations occur in various media [14]. These species are probably collected from certain areas of Brazil which may indicate the effect of environmental factors on the dispersion of mutations in *pncA*. This suggests that the proof of pyrazinamide-resistance in clinical strains of tuberculosis depends on the determination of mutation in each spot of the *pncA* gene. On the other hand, resistant strains are more significantly mutated in two nucleotides of 359 and 374, as suggested by the findings of Dr. Portugal in 2004 [6]. The aforesaid research showed that 42% of these types of mutations are exclusively found in the two mentioned stop codons. This is identically observed in Perdigao (2008) [7]. Tracevska (2004) [8] found five mutated strains in nucleotide 226, the mutation of which was in the form of ACT→CCT [8]. This mutation in 27n led to the transformation of tyrosine amino acid to proline. Mutation in nucleotide 146 was rarely reported in previous studies, as is the case with the current research. Mutation T202G was determined by Louw in 2006 [15] and Lee in 2001 [9], which is consistent with our results. Mutation of nucleotide 212 in the form of A→G was determined by several researchers and we found this mutation in one sample. The current study shows the T→C mutation in nucleotide 515, as suggested by several other researchers in Table 3. 

Sekiguchi (2007) also showed nucleotide 195 (TCC→TCT) variation in some resistant strains [16]. This is a silent mutation which leads to unaltered serine amino acid. The same mutation was found in one case (24n strain), which is consistent with Sekiguchi’s results. 

Sequencing showed alteration in nucleotide 226 (A356C) of one sample (27n). Tracevska (2004) [8] used sequencing to suggest mutation in nucleotide 226 (ACT→CCT) of five strains, which is consistent with our results. In addition to the aforesaid mutations, we discovered new mutations (*G3A* and *T410G)* not found in other literature, as a result of sequencing. Table 3 shows our results. The rapid method presented in this study had a sensitivity and specificity of 66% and 90%, respectively, which is very valuable in the treatment of tuberculosis. One of the features of the proposed method is being an in-house study. It is independent of commercial kits and, expensive devices, and also clarifies all the details of the used compounds and their primers (as opposed to commercial kits), so its costs are low and it is applicable in the laboratory with minimum molecular biology facilities (having a PCR and electrophoresis equipment). As it is shown in Table 3, most of the mutations leading to changes cause bacterial resistance. In this situation, the activity of the Pyrazinamidase enzyme has also been reported as negative and it was proved that the Pyrazinamidase enzyme shows “sensitivity” to the drug. Therefore, it is suggested that biochemical studies on the presence or absence of this enzyme in bacteria isolated from patients should be conducted as a biochemical diagnostic test for the determination of susceptibility to pyrazinamide [2,8].

Resistance to pyrazinamide is developed due to mutation in 561 bp *pncA* and the subsequent deactivation of the pyrazinamide enzyme. Identification of new mutations helps us anticipate the effects of these alterations in protein structures and specify the mechanism of resistance to pyrazinamide. Sequencing is the only safe method to study mutation in the *pncA* gene; however, this method has its own disadvantages, including its expenses and lack of 100% phenotype-genotype adaptation. Nevertheless, the accurate application of sequencing is recommended for finding the possible mutations due to mutations along the *pncA* gene. By discovering all the above mentioned alterations and simulating these alterations in protein dimension, we may take a major step towards the eradication of this fatal disease. 

Based on the results of sequencing of the *pncA* gene, we gained a sense of the amino acid sequence of this gene in the clinical strains studied in this research. Considering the mutated sequences and examination of altered amino acid sequences, the importance of the occurrence of mutations in studied nucleotides can be noticed [2,16]. 

Based on results of Arjomandzadegan , Hosseiny and Khrustalev, it was confirmed that mutations in resistance related genes of *M. tuberculosis* could be caused phenotypic resistance [18,19,20,21] 

**Figure 9:**
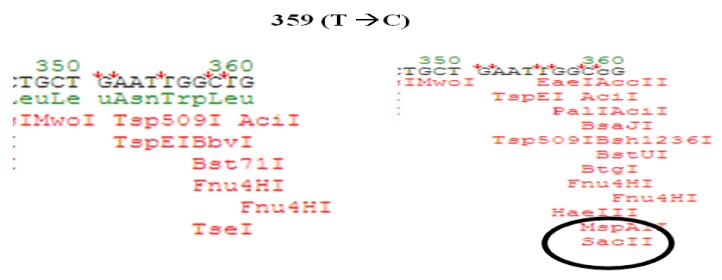
Schematics view from digestion of PCR product fragment by SacII restriction endonuclease (Genetyx Win5.1 software).

Since primers in our sNASP method have been designed to precisely detect the wild type status, it can be said that the final designed technique is a combination of allele specific (APS) and Semi Nested-APS techniques. In this study, the Semi Nested-ASP method was designed for the first time to determine mutations in two nucleotides 374 and 359 in the *pncA* gene. Its relevant explanation is that in nucleotide 359, after the conversion of T to C, hydrophobic leucine amino acid is converted to Proline and in nucleotide 374, after the conversion of T to G valine amino acid is converted to glycine. We used sNASP and RFLP methods for verification of mutation in the 359 and 374 nucleotide. Data showed that the sNASP method could detect mutation, but RFLP does not (Figure 9).

Glycine and proline amino acid are often involved in the formations of turns in the secondary structures of proteins and play an important role in the formation of their third and three-dimensional structures. Following their translation after the occurrence of a mutation, the major changes that occur in the structure of the translated protein will ultimately lead to resistance.

## 5. Conclusions

In this study, the method of the rapid detection of resistance to pyrazinamide was selected after it was compared with a few selected molecular methods and was used to detect pyrazinamide resistance in clinical isolates of *M. tuberculosis*. The sequencing method was also used as the gold standard molecular method and detection of phenotypic resistance using the culture method was used as the gold standard for the resistance.

Mutation occurrence in each spot of the *pncA* gene may lead to drug resistance. Thus, the best method for quick determination of resistance is to determine the overall sequence of the gene. According to the existing literature, the sequence determination results method wherein the entire 561 bp of *pncA* gene undergoes sequencing is considered the only method of detecting mutation in this gene. However, this is a costly method that does not result in 100% phenotype-genotype adaptation. But it is the best way to determine the sequence of all the aforesaid nucleotides in order to determine the resistance genotype and study the probable mutations since it examines the entire gene and specifies the possible mutation anywhere along the gene. Identification of mutations not already reported in *pncA* may turn out to be a major step in more efficient identification of pyrazinamide antibiotic and to defeat the drug resistance of tuberculosis.

## Figures and Tables

**Table 1:** The primer sequences that were used in this study.

Sequence of Primers (5′-3′)		PCR Product Size (bp)
Forward (pnc-8)	GGTTGGGTGGCCGCCGGTCAG	744 bp
Reverse (pnc-11)	GCTTTGCGGCGAGCGCTCCA	
T359C-pnc	AGCCAATTCAGCAGTGGCGTG	418 bp
T374G-pnc	ACGCCGTGCCGCAGCC	433 bp

**Table 2:** Results of sequencing in some samples in this study. R and S indicate resistant and sensitive samples, respectively.

Nucleotide Alteration	Position	Phenotype	Sample ID
G→A	3	R	725
C→T	161	R	571
C→T	195	R	24n
T→G	202	R	734
A→G	212	R	526
T→C	214	R	556
A→C	226	R	27n
T→G	410	R	1228
T→C	515	R	535
none	-	S	4e
none	-	S	36e
none	-	S	11e
none	-	S	16e
none	-	S	1n
none	-	S	2n
none	-	S	3n
none	-	S	5n
none	-	S	6n
none	-	S	7n
none	-	S	9n
none	-	S	10n
none	-	S	11n
none	-	S	13n
none	-	S	15n
none	-	S	16n
none	-	S	19n
none	-	S	21n
none	-	S	22n
none	-	S	23n
none	-	S	24n
none	-	S	25n
none	-	S	26n
none	-	S	29n

**Table 3:** Results of studies examining the *pncA* gene sequence.

Activity of Pyrasinamidase (P: Positive) (N: Negative)	Nucleotide Alteration	Position	Number of Bacterial Samples	Phenotype (R: Resistant, S: Sensitive)	Author	Reference
N	G(3)T	3	1	R	Lee K.W.	[9]
N	A(146)C	146	1	R	Lee K.W.	[9]
?	A(146)C	146	1	R	McCammon M.T.	[10]
N	A(146)T	146	1	R	Marttila H.	[11]
N	R A(146)G	146	1	R	Marttila H.	[11]
?	Del(146)delA	146		R	Somoskovi A.	[12]
	del(161)delC	161	1	R	Lee K.W.	[9]
N	C(161)A	161	1	R	Barco P.	[14]
?	C(161)T	161	1	R	Louw G.E.	[15]
N	C(161)T	161	6	R	Sekiguchi J.I.	[16]
?	R C(161)T	161	1	R	McCammon M.T.	[10]
?	C(195)T	195	2	S	Somoskovi A.	[12]
?	C(195)T	195	1	S	McCammon M.T.	[10]
?	T(202)C	202	1	R	Somoskovi A.	[12]
?	T(202)C	202	3	R	Louw G.E.	[15]
N	T(202)C	202	2	R	Lee K.W.	[9]
?	T(202)G	202	7	R	Louw G.E.	[15]
N	T(202)G	202	1	R	Lee K.W.	[9]
?	A(212)G	212	1	R	Somoskovi A.	[12]
?	A(212)G	212	3	R	Louw G.E.	[15]
N	T(214)C	214	1	R	Hirano K.	[17]
N	A(410)C	410	1	R	Lee K.W.	[9]
?	T(515)C	515	3	R	Somoskovi A.	[12]
N	T(515)C	515	1	R	Lee K.W.	[9]

## Abbreviations

CDS	Coding Sequence
MTB	*Mycobacterium tuberculosis*
RFLP	Restriction fragment of length polymorphism
sNASP	Semi-nested allele specific PCR

## References

[1] World Health Organization (WHO) (2013). Global Tuberculosis Report 2013. WHO Library Cataloguing-In-Publication Data.

[2] Pourhajibagher M., Nasrollahi M. (2012). Drug Resistance in *Mycobacterium tuberculosis* Isolates to Isoniazid and Rifampin. J. Babol. Univ. Med. Sci..

[3] Palomino J.C., Martin A. (2014). Drug Resistance Mechanisms in *Mycobacterium tuberculosis*. Antibiotics.

[4] Zhang Y., Heym B., Allen B., Young D., Cole S. (1992). The Catalase. Peroxidase gene and Isoniazid Resistance of *Mycobacterial tuberculosis*. Nature.

[5] Walsh P.S., Metzger D.A., Higuchi R. (1991). Chelex 100 as a medium for simple extraction of DNA for PCR-based typing from forensic material. BioTechniques.

[6] Portugal I., Barreiro L., Moniz-Pereira J., Brum A.L. (2004). *pncA* mutations In Pyrazinamide-Resistant *Mycobacterium tuberculosis* Isolates in Portugal. Antimicrob. Agents Chemother..

[7] Perdigão J., Macedo R., João I., Fernandes E., Brum L., Portugal I. (2008). Multidrug-Resistant Tuberculosis in Lisbon, Portugal: A Molecular Epidemiological Perspective. Microb. Drug Resist..

[8] Tracevska T., Nodieva A., Skenders G. (2004). Spectrum of *pncA* mutations in Multidrug-Resistant *Mycobacterium tuberculosis* Isolates Obtained In Lativia. Antimicrob. Agents Chemother..

[9] Lee K.W., Lee J.M., Jung K. (2001). Characterization of *pncA* Mutations of Pyrazinamide-Resistant *Mycobacterium tuberculosis* in Korea. J. Korean Med. Sci..

[10] McCammon M.T., Gillette J.S., Thomas D.P., Ramaswamy S.V., Graviss E.A., Kreiswirth B.N., Vijg J., Quitugua T.N. (2005). Detection of *rpoB* mutations associated with rifampin resistance in *Mycobacterium tuberculosis* using denaturing gradient gel electrophoresis. Antimicrob. Agents Chemother..

[11] Marttila H.J., Marjamaki M., Vyshnevskaya E., Vyshnevskiy B.I., Otten T.F., Vasilyef A.V., Viljanen M.K. (1999). *pncA* mutations in pyrazinamide-resistant *Mycobacterium tuberculosis* isolates from northwestern Russia. Antimicrob. Agents Chemother..

[12] Somoskovi A., Wade M.M., Sun Z., Zhang Y. (2004). Iron enhances the antituberculous activity of pyrazinamide. J. Antimicrob. Hemother..

[13] Sheen P., Ferrer P., Gilman R.H., López-Llano J., Fuentes P., Valencia E., Zimic M.J. (2009). Effect of pyrazinamidase activity on pyrazinamide resistance in *Mycobacterium tuberculosis*. Tuberculosis.

[14] Barco P., Cardoso R.F., Hirata R.D.C., Leite C.Q.F., Pandolfi J.R., Sato D.N., Shikama M.L., de Melo F.F.z., Mamizuka E.M., Campanerut P.A.Z. (2006). *pncA* mutations in pyrazinamide-resistant *Mycobacterium tuberculosis* clinical isolates from the southeast region of Brazil. J. Antimicrob. Chemother..

[15] Louw G.E., Warren R.M., Donald P.R., Murray M.B., Bosman M., van Helden P.D., Young D.B., Victor T.C. (2006). Frequency and implications of pyrazinamide resistance in managing previously treated tuberculosis patients. Int. J. Tuberc. Lung Dis..

[16] Sekiguchi J.I., Nakamura T., Miyoshi-Akiyama T., Kirikae F., Kobayashi I., Augustynowicz-Kopeć E., Zwolska Z., Morita K., Suetake T., Yoshida H. (2007). Development and evaluation of a line probe assay for rapid identification of *pncA* mutations in pyrazinamide-resistant Mycobacterium tuberculosis strains. J. Clin. Microbiol..

[17] Hirano K., Takahashi M., Kazumi Y., Fukazawa Y., Abe C. (1998). Mutation in *pncA* is a major mechanism of pyrazinamide resistance in *Mycobacterium tuberculosis*. Tuberc. Lung Dis..

[18] Arjomandzadegan M., Owlia P., Ranjbar R., Farazi A., Masume S., Maryam S., Ghaznavi-Rad E., Surkova L., Titov L. (2011). Prevalence of mutations at codon 463 of *katG* gene in MDR and XDR clinical isolates of *Mycobacterium tuberculosis* in Belarus and application of the method in rapid diagnosis. Acta Microbiol. Immunol. Hung..

[19] Hosseini H., Fooladi A.A.I., Arjomandzadegan M., Emami N., Bornasi H. (2014). Genetics study and transmission electron microscopy of pili in susceptible and resistant clinical isolates of *Mycobacterium tuberculosis*. Asian Pac. J. Trop. Biomed..

[20] Arjomandzadegan M., Titov L.P., Surkova L.K., Farnia P., Sheikholeslami F., Owlia P., Eshghinejad A., Farazi A.A., Eshrati M., Kahbazi M. (2012). Determination of principal genotypic groups among susceptible, MDR and XDR clinical isolates of *Mycobacterium tuberculosis* in Belarus and Iran. Tuberk. Toraks.

[21] Khrustalev V.V., Arjomandzadegan M., Barkovsky E.V., Titov L.P. (2012). Low rates of synonymous mutations in sequences of *Mycobacterium tuberculosis GyrA* and *KatG* genes. Tuberculosis.

